# Heterogeneity in geographical trends of HIV epidemics among key populations in Pakistan: a mathematical modeling study of survey data

**DOI:** 10.7189/jogh.08.010412

**Published:** 2018-06

**Authors:** Dessalegn Y Melesse, Leigh Anne Shafer, Faran Emmanuel, Tahira Reza, Baseer K Achakzai, Sofia Furqan, James F Blanchard

**Affiliations:** 1 Centre for Global Public Health, Department of Community Health Sciences, University of Manitoba, Winnipeg, Manitoba, Canada; 2 Department of Internal Medicine, University of Manitoba, Winnipeg, Manitoba, Canada; 3 Centre for Global Public Health, Islamabad, Pakistan; 4 National AIDS Control Program, National Institute of Health, Islamabad, Pakistan

## Abstract

**Background:**

Assessing patterns and trends in new infections is key to better understanding of HIV epidemics, and is best done through monitoring changes in incidence over time. In this study, we examined disparities in geographical trends of HIV epidemics among people who inject drugs (PWIDs), female sex workers (FSWs) and *hijra*/transgender/male sex workers (H/MSWs), in Pakistan.

**Methods:**

The UNAIDS Estimation and Projection Package (EPP) mathematical model was used to explore geographical trends in HIV epidemics. Four rounds of mapping and surveillance data collected among key populations (KPs) across 20 cities in Pakistan between 2005-2011 was used for modeling. Empirical estimates of HIV prevalence of each KP in each city were used to fit the model to estimate prevalence and incidence over time.

**Results:**

HIV incidence among PWIDs in Pakistan reached its peak in 2011, estimated at 45.3 per 1000 person-years. Incidence was projected to continue to rise from 18.9 in 2015 to 24.3 in 2020 among H/MSWs and from 3.2 in 2015 to 6.3 in 2020 among FSWs. The number of people living with HIV in Pakistan was estimated to steadily increase through at least 2020. HIV incidence peak among PWIDs ranged from 16.2 in 1997 in Quetta to 71.0 in 2010 in Faisalabad (per 1000 person-years). Incidence among H/MSWs may continue to rise through 2020 in all the cities, except in Larkana where it peaked in the early 2000s. In 2015, model estimated incidence among FSWs was 8.1 in Karachi, 6.6 in Larkana, 2.0 in Sukkur and 1.2 in Lahore (per 1000 person-years).

**Conclusions:**

There exists significant geographical heterogeneity in patterns and trends of HIV sub-epidemics in Pakistan. Focused interventions and service delivery approaches, different by KP and city, are recommended.

Pakistan is facing concentrated HIV epidemics among key populations (KPs), specifically people who inject drugs (PWIDs) and sex workers (SWs) [1-5]. KPs in Pakistan, particularly female, male and transgender sex worker populations (FSWs, MSWs and HSWs), engage in transactional sex underground, limiting their access to services and potentially subjecting them to harassment and violence [6-9]. The HIV epidemic is well established among PWIDs, and is perhaps expanding among SWs in Pakistan. It has been suggested that the expansion of HIV to SWs may be related to structural and sociocultural aspects of the KPs, such as cultural stigma, poor knowledge about how to prevent HIV, low literacy and inadequate access to services [10,11].

Empirical evidence obtained from integrated biological and behavioural surveillance (IBBS) data suggested that HIV prevalence among PWIDs increased from an estimated (unweighted) 11% in 2005 to 27% by 2011 [12-15]. Evidence from these IBBS data also indicated that prevalence among HSWs, MSWs and FSWs in 2011 was estimated at 5.2%, 1.6% and 0.6% [12-15]. Studies have shown that SWs in Pakistan are at a higher risk of HIV exposure due to their interaction with PWIDs through sex and/or needle sharing [12-15]. Although vulnerability varies by KP and city, there is growing concern that the virus may be expanding to sexual contacts of PWIDs, particularly SWs, as well as to the general population through sexual networks of SWs and their clients [12,16-20].

Assessing incidence patterns helps to better understand HIV epidemic trends. Monitoring global changes in incidence is no longer sufficient, and more disaggregated and fine-grained assessments have been suggested for improved estimation of HIV epidemics [21]. Incidence as opposed to prevalence may generate insights about potential factors contributing to the epidemics. However, it is prevalence data that is often available and used in Pakistan to inform policy and guide the design of intervention programs. Although prevalence data are currently available in certain cities and years in Pakistan [12,17,20,22-25], these empirical prevalence estimates are not globally available across all of Pakistan, and are often limited to estimates only among certain higher risk groups [24]. Furthermore, there is limited data on the change in number of new HIV infections over time on a more disaggregate level.

Using mathematical modeling, we examined geographic disparities in the trends and emerging patterns of HIV sub-epidemics using model estimated prevalence and incidence among KPs in Pakistan. This study provides disaggregated assessments of the levels and trends of the HIV epidemics in Pakistan.

## METHODS

### Study settings

Between 2005 and 2011, the Canada-Pakistan HIV/AIDS Surveillance Project (HASP) collected four rounds of IBBS data across Pakistan [12-15,26]. The number of cities (up to 20) and KPs surveyed in each round varied. Details on cities included can be found in previous reports [12-15]. Maps of cities surveyed in 2005 and 2011, depicting relative prevalence among KPs, can be found elsewhere [27].

### Study population

The KPs surveyed were PWIDs, HSWs, MSWs and FSWS. MSWs and HSWs refer to men aged 13 and 15 or older, respectively, and FSWs refers to women aged 15 or older who sell sex in exchange for money or gifts. Many MSWs start sex work at a younger age than FSWs or HSWs, and therefore the age limit for inclusion in MSWs sample was lower. Most h*ijras* are transgender individuals who are born males and often cross-dress in feminine attire, only a few having been born with intersex variations, and some having undergone surgical sex change [28-30]. They form a distinct sociocultural group unique to South Asia, and are considered to be a third gender [28-30]. PWIDs refers to persons aged 18 or older who had injected drugs in the past six months. Because HSWs and MSWs are both biologically males and have overlapping of networks, including client networks, IBBS data among these KPs were collected together except in the fourth round conducted in 2011. The difference age cut-offs in the inclusion criteria is based on anecdotal evidence and age distribution of each KP in subsequent IBBS. It is also important to note that inclusion and exclusion criteria were ascertained through a broad National consultative process in which all stakeholders along with National and Provincial AIDS Control Programs participated.

### Data collection

Each round of IBBS began with an in-depth network mapping exercise to estimate the size, distribution and operational typology of PWIDs and SWs in targeted cities [12-15], except the third round (in 2008) where the second round mapping (in 2006/7) was used as a basis to recruit study samples [13]. Details of the mapping can be found elsewhere [12-15,31]. Cities were selected based on anecdotal evidence of high risk activity, the presence of multiple KPs, and the geographical accessibility of the area. Following mapping, a representative sample of each KP was drawn in each city. These cities were identified before the start of each round of surveillance through a broad National consultative process in which all stakeholders along with National/Provincial AIDS Control Programs participated.

Various techniques were utilized to recruit representative KP samples. For instance, MSWs were recruited through respondent driven sampling, while HSWs were recruited through network sampling whereby *gurus*, “retired” HSWs, were selected randomly from a list compiled from the previous mapping results and asked to recruit eligible subjects. Street-based FSWs were recruited using time-location cluster sampling and PWIDs were recruited using multistage cluster sampling. Data was then collected from samples of each KP in each city by trained interviewers using structured questionnaires. Data from FSWs were not collected in the third round (2009) because of low HIV prevalence observed in 2006/7. Stratified by survey round, city, and KP, the sample size was greater than 350 in more than 86% of the strata (average sample size per strata ~ 400). Further details on the methods and techniques of mapping, sampling and data collection have been described previously [12-15,31].

### Data analysis

#### Statistical analysis

Empirical HIV prevalence and 95% confidence intervals (CI) in each round, city and KP were computed. Sampling weights based on the respective estimated population sizes of each KP in each city were utilized when appropriate to take into account the stratified sampling design.

Using empirical HIV prevalence estimates from each survey round as inputs, mathematical modeling was performed to estimate and project the prevalence of HIV among each KP in each city and year (even those years not surveyed). Along with estimated HIV prevalence by KP, the model provided estimates of the total number of people living with HIV, the number of people who died from HIV, and the population size of the HIV uninfected people over time. Using these model outputs, we then estimated HIV incidence. The combined data of MSWs and HSWs (abbreviated here as H/MSWs) were used for analysis because they are similar in terms of their behavior and risk to HIV acquisition. Both groups were born as biological males and have male clients; however, HSWs are transgender.

#### Mathematical modeling

We used the UNAIDS Estimation and Projection Package model (EPP) to estimate and project HIV prevalence [32-34]. The EPP model, a deterministic compartmental model which estimates HIV prevalence over time by fitting to empirically estimated HIV prevalence at certain time points, has been described previously [33-35]. Further details on our modeling using EPP, including sources and method used to compute input parameters to fit the model, model scenarios, plausible estimates of prevalence, and sensitivity analysis has been described previously [24].

EPP allows separate model fitting for different subpopulations of different cities and may combine them for aggregate prevalence estimates. Model estimates of prevalence were assessed by sensitivity analyses. In sensitivity analyses, we ran the model multiple times, fit to lower and upper bounds of the 95% CIs of empirically estimated HIV prevalence of each city. In addition to city-specific HIV prevalence, EPP generates an estimated national prevalence by combining each KP subpopulations’ and cities’ estimated prevalence. Given that the HIV epidemic in Pakistan is largely concentrated among KPs in urban and semi-urban areas, this study assumed that HIV prevalence in rural Pakistan, while not non-existent, is almost negligible [36,37].

In this study, we used data from all 20 cities surveyed to produce national (among KP) and all-KP estimates of HIV incidence and prevalence. In our disaggregated analyses, however, we focused on the 10 cities that were represented in at least two rounds of the IBBS in each KP: Faisalabad, Hyderabad, Karachi, Lahore, Larkana, Multan, Peshawar, Quetta, Sargodha, and Sukkur.

## RESULTS

Analysis was based on 43 522 individuals (27.8% FSWs, 33.1% PWIDs, and 39.1% H/MSWs) from 20 Pakistani cities. The mean age of PWIDs, FSWs and H/MSWs was 32.5 (standard deviation, SD = 8.4), 27.3 (SD = 6.6) and 24.5 (SD = 6.4) years, respectively. The average number of years that they had been involved in high-risk activities (ie, in sex work or drug injection) by the time of their interview was 5.1 (PWIDs), 5.4 (FSWs) and 8.3 (H/MSWs) years.

### Estimated prevalence of HIV

**Figure 1:**
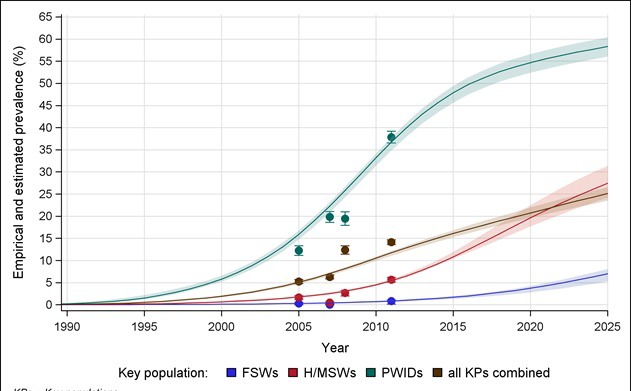
Empirical and model estimated prevalence of HIV among key populations in Pakistan. PWIDs – people who inject drugs, FSWs – female sex workers, H/MSWs – hijra/transgender/male sex workers, KPs – key populations. Dots/bars indicate empirical estimates/95% confidence interval of prevalence. Shades regions indicate the range of estimated incidence.

**Figure 2:**
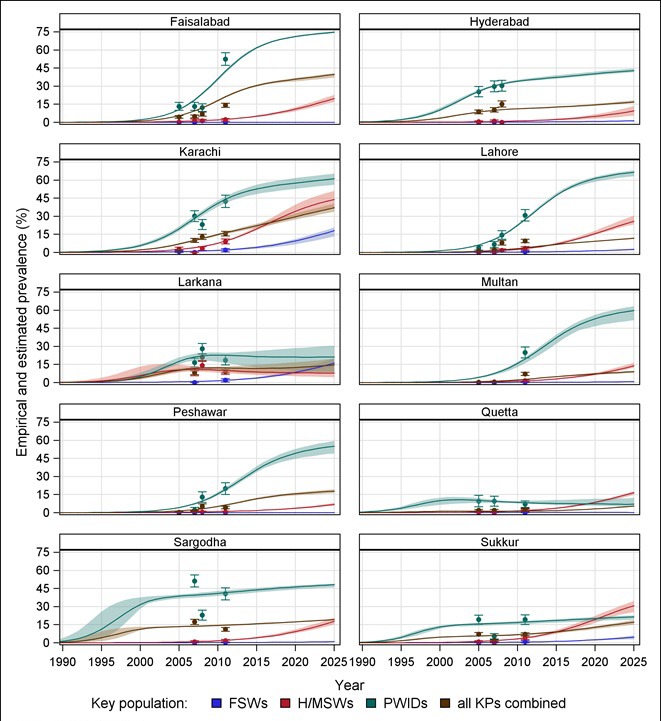
Empirical and model estimated prevalence of HIV among each key population in each city. PWIDs – people who inject drugs, FSWs – female sex workers, H/MSWs – hijra/transgender/male sex workers, KPs – key populations. Dots/bars indicate empirical estimates/95% confidence interval of prevalence. Shades regions indicate the range of estimated incidence.

Model estimates suggest that HIV prevalence in the adult population will continue to rise in Pakistan, increasing from 0.05% (plausible range, 0.04-0.06%) in 2015 to 0.07% (plausible range, 0.06-0.08%) by 2020. Prevalence among all KPs in Pakistan was projected to increase from 16.1% (plausible range, 15.2-16.8%) in 2015 to 20.8% (plausible range, 19.6-21.9%) in 2020, although our results suggest that the burden of the epidemics will vary considerably between KPs (**Figure 1**) and cities (**Figure 2**). More than half of PWIDs (54.7%, plausible range: 52.4-56.9%) and nearly one-fifth of H/MSWs (19.6%, plausible range: 17.7-22.4%) may be living with HIV by 2020. By the same year, HIV prevalence among FSWs may reach 3.5% (plausible range, 2.8-4.3%).

Among FSWs, estimated prevalence in all cities is estimated to rise throughout the projection period (**Figure 2**). Among H/MSWs, estimated prevalence rises throughout the projection period, except in Larkana, where it peaked around 2007, estimated at 10.9%. Estimated HIV prevalence among PWIDs in Quetta might have peaked in the early 2000s, followed by a relatively constant prevalence around 8.1%. In all other cities, prevalence among PWIDs is estimated to continue rising throughout the projection period.

### Estimated incidence of HIV

**Figure 3:**
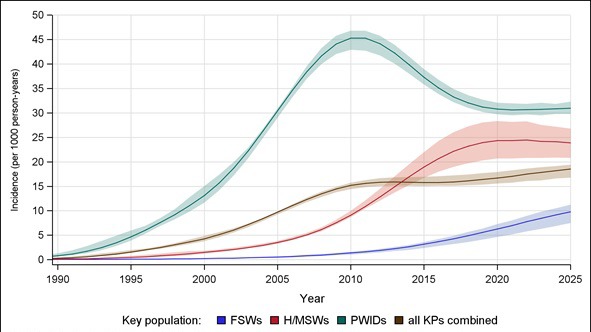
Estimated HIV incidence among key populations in Pakistan. PWIDs – people who inject drugs, FSWs – female sex workers, H/MSWs – hijra/transgender/male sex workers, KPs – key populations. Shaded regions indicate the range of estimated incidence.

In the adult populations of Pakistan, model-estimated HIV incidence peaked in 2015, at 0.06 per 1000 person-years (plausible range, 0.05-0.07). By contrast, HIV incidence among all KPs combined was projected to increase, rising from 15.8 (plausible range, 15.1-16.9) in 2015 to 16.7 (plausible range, 15.5-17.9) per 1000 person-years by 2020 (**Figure 3**). This increase is a result of rising incidence among H/MSWs and FSWs. In Pakistan, HIV incidence among PWIDs peaked in 2011, estimated at 45.3 new infections per 1000 person-years (range, 42.7-46.8), and is projected to fall to 30.8 (plausible range, 29.7-32.3) by 2020, before stabilizing. By contrast, estimated incidence among H/MSWs has increased from 9.1 new infections per 1000 person-years (plausible range, 8.6-10.0) in 2010 to 18.9 (plausible range, 16.7-22.0) in 2015 and projected to continue rising to 24.3 (plausible range, 20.7-28.3) by 2020. Although incidence among FSWs is lower than among other KPs, it is estimated to increase from 1.4 new infections per 1000 person-years (plausible range, 1.0-1.7) in 2010 to 6.2 (plausible range, 4.6-7.3) by 2020.

**Figure 4:**
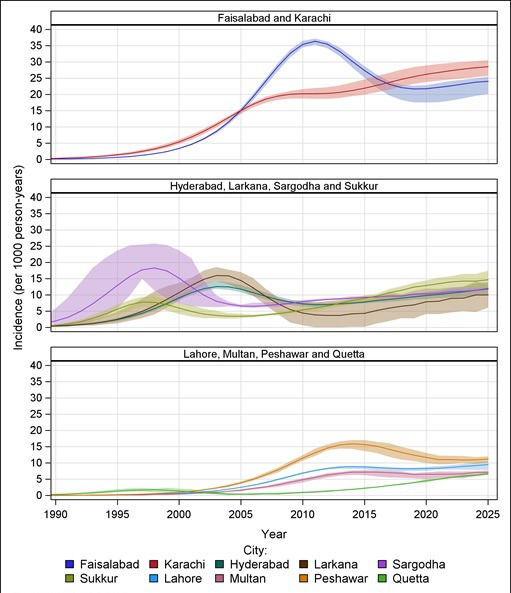
Estimated HIV incidence among all key populations combined in each city. KPs – key populations. Shaded regions indicate the range of estimated incidence.

Similar to the variation in prevalence trends by city, our modeling suggests three distinctively different incidence patterns among all KPs by city (**Figure 4**). These geographical disparities in the patterns of the HIV epidemic are related to: the timing that the epidemic started, the timing that the epidemic is likely to have peaked, the number of new infections at a given time, and the change in the trajectory of HIV transmission overtime. The HIV incidence among all KPs combined is highest in Karachi and Faisalabad. Incidence peaked early in Hyderabad, Larkana, Sargodha, and Sukkur, but after a period of decline, it began to rise again. In Sukkur in particular, current HIV incidence among all KPs is estimated to be higher than it was at its early peak, and is estimated to continue rising beyond the year 2020. Incidence in Lahore, Multan, Peshawar, and Quetta is lower and with less pronounced peaks and troughs than in the other cities.

**Figure 5:**
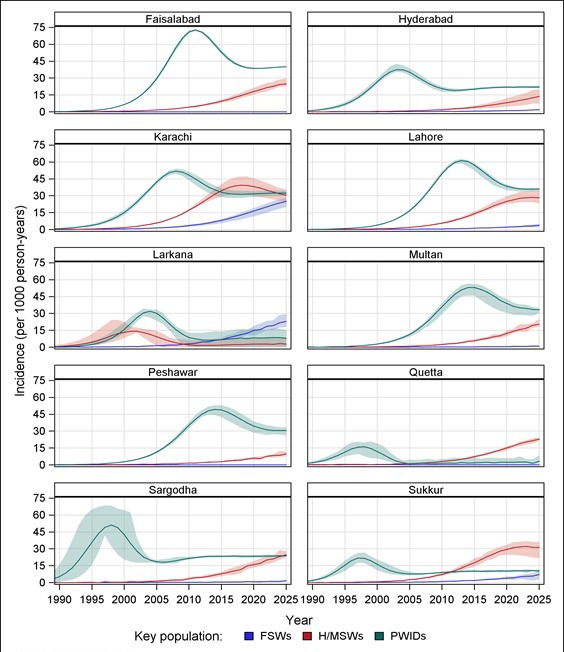
Estimated HIV incidence among key populations within each city. PWIDs – people who inject drugs, FSWs – female sex workers, H/MSWs – hijra/transgender/male sex workers, KPs – key populations. Shaded regions indicate the range of estimated incidence.

At a further disaggregated level, each KP has geographically different HIV incidence patterns (**Figure 5**). HIV incidence among PWIDs in each city peaked on or before 2015, with peak incidence ranging from 16.2 (plausible range, 7.6-19.8) in 1997 in Quetta to 71.0 (plausible range, 69.1-72.1) in 2010 in Faisalabad (per 1000 person-years). Incidence among H/MSWs was projected to continue to increase through 2020 in all cities, except in Larkana. HIV epidemics began to emerge among FSWs in the past 15 years, and will likely continue rising in most cities. By 2020, incidence among FSWs is estimated at 16.4 (plausible range, 11.6-19.0) in Karachi, 14.9 (10.3-18.7) in Larkana, 4.0 (plausible range, 2.6-5.4) in Sukkur and 2.1 (plausible range, 1.6-2.5) in Lahore (per 1000 person-years) (to better distinguish trends, incidence among FSWs is presented on a different scale in Figure S1 in **Online Supplementary Document(Online Supplementary Document)**).

## DISCUSSION

Pakistan is experiencing an increase in HIV infections among KPs, particularly among H/MSWs and FSWs. The epidemic continues to vary considerably by KP and city, and the time lag in the mergeing trends of the epidemics among KPs perhaps suggesting multiple interconnected sub-epidemics within the country. In addition to heterogenous HIV incidence and prevalence trends, there is also variation in the mean duration that each KP were engaged in high-risk activity. The youngest KP (H/MSWs, mean age 24.5 years) have the longest duration of activity (mean 8.3 years).

PWIDs, followed by H/MSWs, remain most severely affected KPs, with nearly 1 in every 2 PWIDs and 1 in every 5 H/MSWs living with HIV by 2020 (**Figure 1**). Previously estimated rising HIV prevalence among FSWs in Karachi, Larkana and Lahore will likely be seen in most cities across Pakistan [24]. While HIV incidence among all KPs in Pakistan is estimated to increase through 2020, it will vary by KP. HIV incidence among SWs, for example, is estimated to emerge and remain increasing, while incidence among PWIDs possibly peaked in 2011. HIV incidence among H/MSWs may peak in the early 2020s. This highlights public concerns that HIV incidence might be spreading from PWIDs to the rest of the population through sexual networks [12-15,26].

### HIV prevalence and incidence in the general population

HIV prevalence in the general adult population is estimated to rise from a range of 0.04-0.06% in 2015 to 0.06-0.08% by 2020. Although we only used empirically estimated prevalence from KPs as input for our modeling, our model estimates of HIV prevalence in the adult population of Pakistan in 2011 is similar to the empirical prevalence estimate obtained from a large national population-based Antenatal Care (ANC) study conducted in 2011 [26]. Also, the 2015 model estimates of HIV prevalence in the general population are similar to those reported previously by UNAIDS [36,37].

HIV incidence in the general adult population of Pakistan peaked in 2015, estimated at 0.062 per 1000 person-years. This contrasts with worldwide HIV incidence which peaked around 1997 [21,38]. Our estimate of the number of new infections in 2015 (10,756; plausible range: 8049-14,414) is between the slightly higher estimate reported by UNAIDS (approximately 11 500) [37] and the somewhat lower estimate provided by the Global Burden of Disease Study (8,550) [21]. The difference with the UNAIDS estimate may be partly due to unavailability in our data on HIV prevalence from sex worker clients and low-risk populations such as rural Pakistan, the age and estimated size of key population used, and the lack of data on antiretroviral therapy (ART) coverage.

### Social and sexual networking patterns and HIV sub-epidemics in Pakistan

The emerging patterns of HIV epidemics among KPs in Pakistan is consistent with the patterns observed in other Asian countries [3,39,40], whereby HIV transmission begins among PWIDs and subsequently spreads to SWs and their clients. In most cities in Pakistan, rising HIV incidence first occurred among PWIDs before or early in the 1990s, followed by H/MSWs later in the 1990s, and then FSWs early in the 2000s. Following global trends in HIV epidemic, whereby increased migration precedes the spread of HIV infection among PWIDs [41], the HIV epidemic in Pakistan might have been introduced by migrant workers returned from Gulf states [42,43], and then spread to KPs [44,45]. It has been highlighted high prevalence of HIV among returned migrant workers from Gulf countries, with nearly 61-86% of reported HIV cases in any given year during the 1996-1998 period [42], followed by reported outbreaks of HIV infections among PWIDs in subsequent years [44,45].

Though HIV incidence among PWIDs declined after 2011, HIV prevalence continued to rise in most cities. Among all KPs combined in Pakistan, both incidence and prevalence is estimated to continue increasing. This is due to the rapid upsurge of HIV transmission among H/MSWs followed by FSWs. What are the possible mechanisms that explain the emergence of SW epidemics following those of PWIDs in Pakistan? One explanation is the extent to which SW populations mix with PWIDs, which impacts HIV transmission risk vulnerability through overlapping social and sexual networks. The greater the overlap between SWs and PWIDs, the quicker HIV reaches the wider population. Evidence from IBBS data has suggested that there is a significant proportion of KPs with overlapping risk behaviour, also called “bridge” populations – individuals who in this case engage in multiple risk behaviours (ie, through having sex and/or needle-sharing with a different KP) [12-15,24]. SWs who interact sexually with PWIDs increase their HIV risk in two ways – they are having sex with other higher risk populations, and they have higher odds of injecting drugs themselves. Our previous epidemiological study indicated that H/MSWs have higher odds of interacting with PWIDs through sex and/or needle sharing than do FSWs [46]. This implies that H/MSWs are more vulnerable than FSWs to HIV transmission from PWIDs. Furthermore, because male-to-male HIV transmission is more efficient than both male-to-female and female-to-male transmission, H/MSWs are at higher risk of acquiring HIV from their male sexual partners who inject drugs. Therefore, in Pakistan, where the epidemic is predominantly driven by PWIDs, H/MSWs are more vulnerable than FSWs as a result of their interaction with PWIDs [46]. The H/MSWs are presumably at a higher risk of an emergent epidemic, followed by FSWs.

### HIV epidemic in Karachi

Karachi is currently experiencing escalated HIV incidence among KPs. Although this is largely due to a rapid upsurge of new infections among both H/MSWs and FSWs, all three KPs might have substantial contributions to sustain steadily increasing incidence in Karachi through at least 2020. HIV incidence among H/MSWs in Karachi likely surpassed that of among PWIDs in 2015, and will remain higher through the early 2020s. Given the increasing trend of HIV incidence among FSWs in Karachi, the epidemic may become as explosive among FSWs as among the other two KPs in the future. This study is consistent with previous work suggesting that HIV incidence among PWIDs in Karachi may have risen rapidly in early 2000s, and perhaps the epidemic subsequently spilled over into H/MSWs followed by FSWs [17].

### HIV epidemic in Faisalabad

Faisalabad is similar to Karachi in terms of high HIV prevalence and incidence, which may be largely due to increased incidence among PWIDs followed by H/MSWs (**Figure 5**). Patterns among KPs, however, differ. Unlike in Karachi, the rapidly increasing incidence among H/MSWs in Faisalabad is likely to peak around 2018. New infections among FSWs have negligible effect on the overall incidence among KPs in the Faisalabad.

### HIV epidemics in Larkana, Quetta, Sukkur, Hyderabad and Sargodha

Unlike other cities, where PWIDs have been the driving force in HIV incidence, SWs have had a major role in incidence in Larkana, Quetta and Sukkur since 2010. That said, the SW group driving the epidemic varies considerably between these cities. The upsurge of new infections in Larkana remains mostly among FSWs, while the epidemics in Quetta and Sukkur are largely driven by H/MSWs. HIV incidence among FSWs in Larkana, and H/MSWs in Quetta and Sukkur, might have surpassed that of PWIDs in the early 2010s.

The HIV epidemics among PWIDs in these cities (Larkana, Quetta, Sukkur, Sargodha and Hyderabad) peaked early and has remained relatively low and stable since 2005. This may imply that HIV infections among PWIDs are circulating among clusters of individuals, but the epidemic is likely expanding into the SW population through sexual networks. We may speculate that heterosexual networking in Larkana and homosexual networking in the other cities may have played a role in the spread of the epidemics among SWs.

### HIV epidemics in Lahore, Multan and Peshawar

HIV incidence in Lahore, Multan and Peshawar is largely driven by PWIDs, followed by H/MSWs. HIV incidence among all KPs in these cities may have started rising more recently than in other cities, and may have reached their peaks later, in the mid-2010s.

### Limitations

EPP is not a perfect model, and we share limitations that were discussed in previous studies [34,35,47]. In addition, the absence of data on ART distribution, data on HIV prevalence among clients of SWs, and data on HIV prevalence among adults with unknown higher risk of HIV transmission, were limitations of this study. Although scenarios were considered to capture potential variations in the estimation and projection of HIV prevalence and incidence that may arise due to variation in duration of time that persons in each KP were engaged in high-risk behaviour, it was difficult to make accurate estimates of these durations. Finally, the range between the minimum and maximum prevalence and incidence consistently increased over time through 2025, which underlines the uncertainty in estimating this far into the future. Thus, it is our results are not definitive, but suggest a good description of the underlying aggregate and disaggregate HIV dynamics in Pakistan. National level estimations and projection based on the assumption of HIV prevalence in rural Pakistan as negligible during surveys may not reflect the true nature of the epidemic in subsequent years, and thus results must be interpreted cautiously.

## CONCLUSIONS

The HIV epidemic in Pakistan is comprised of multiple sub-epidemics among KPs, with considerable variations in geographical trends. Both sexual and injection networks in Pakistan may have uniquely contributed to the spread of the epidemic from PWIDs to SWs. The combination of high HIV prevalence among PWIDs along with emerging epidemics among SWs calls for an urgent response and implementation of targeted interventions and service delivery approaches in Pakistan. Attention should be paid to drug injection and sexual mixing patterns of sex workers with PWIDs, largely because sexual networks are a gateway through which HIV could spread to the general population. In order to explore what specific interventions may have the largest impact on mitigating the HIV sub-epidemics in Pakistan, research is warranted for a more in-depth understanding of underlying social and structural factors along the pathway to HIV risk vulnerabilities among SWs, as well as in the general population.
