# Supplementary material for: Heterogeneity in geographical trends of HIV epidemics among key populations in Pakistan: a mathematical modeling study of survey data
Source: J Glob Health. 2018 May 10;8(1):010412. doi: 10.7189/jogh.08.010412 (PMC5944903; doi:10.7189/jogh.08.010412)
Supplement: Online Supplementary Document [file jogh-08-010412-s001.pdf]

Online Supplementary Document

Melesse et al. Heterogeneity in geographical trends of HIV epidemics among key populations in Pakistan: a mathematical modeling study of survey data

J Glob Health 2018;8:010412

Supplementary Figure 1. Estimated incidence among FSWs by city

The results presented in this supplemental figure is the same as in Figure 5, except the incidence on the supplemental figure is presented on a different scale to better distinguish trends of HIV incidence among FSWs.

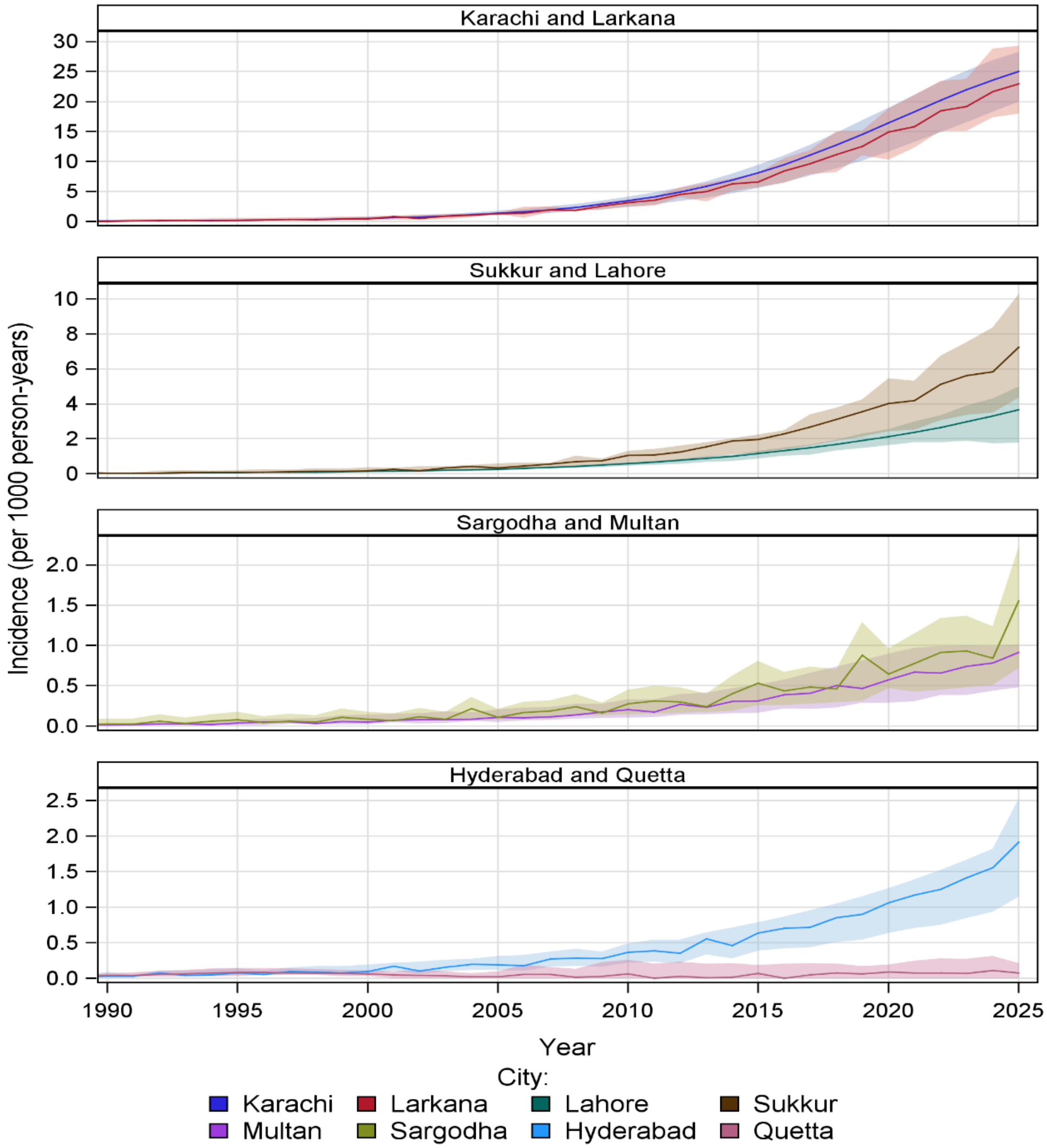

Shaded region indicates the range of estimated incidence among FSWs.
FSWs = Female sex workers.
**Note: 1. Faisalabad and Peshawar are not included as they have zero empirical prevalence.*
2. Different scales were used on the vertical (incidence) axis.
